# Barriers for using amoxycillin dispersible tablet in pediatric pneumonia treatment in Bangladesh

**DOI:** 10.1093/eurpub/ckac129.168

**Published:** 2022-10-25

**Authors:** M Shah, S Ahmed, S Rana, G Mothabbir, J Islam, S Islam

**Affiliations:** 1 Global Health, Save the Children US, Washington DC, USA; 2 Health and Nutrition, Save the Children International, Dhaka, Bangladesh; 3 NNHP & IMCI, Ministry of Health, Dhaka, Bangladesh

## Abstract

World Health Organization recommends guideline for integrated management of childhood illnesses (IMCI) where Amoxicillin Dispersible Tablet (DT) appears as the first drug of choice for treating childhood pneumonia. The Government of Bangladesh adopted the IMCI strategy in 1998, and scaled it up nationwide by 2014. But, even today, the use of Amoxicillin DT, either in public or private sector, for managing childhood pneumonia is a rare event in Bangladesh. We conducted this exploratory study to understand the existing barriers, both in public and private sector, those have influences on reduced availability of Amoxicillin DT and non-compliance of health service providers to follow IMCI guideline by using Amoxicillin DT for treating a child with pneumonia, in Bangladesh. We conducted desk review of relevant strategy and policy documents, key informant interviews with 19 key individuals from Ministry of Health and national / international NGOs. Collected information were analyzed and interpreted using thematic analysis method. Identified barriers through this study pointed to inadequate policy level focus on IMCI implementation, non-inclusion of Amoxicillin DT in the national essential drug list, single source of Amoxicillin DT producing pharmaceutical in the country coupled with bureaucracy and procurement procedural complexity, lack of training of health service providers and abundant availability of antibiotic over the counter. Study respondents recommended for policy level strengthening of IMCI program, increasing coverage of training for health care provider, including practicing pediatricians both at public and private sectors, facilitating production and procurement procedures and prohibiting antibiotic sell over the counter.

**Key messages:**

• Facilitation of production and procurement procedure coupled with enforcement of law prohibiting antibiotics availability over the counter are urgent needs.

• Policy level support emphasizing full compliance of service providers for quality of implementation of IMCI program in Bangladesh (and similar other settings) is also important.

